# Platinum corrosion products from electrode contacts of human cochlear implants induce cell death in cell culture models

**DOI:** 10.1371/journal.pone.0196649

**Published:** 2018-05-15

**Authors:** Kirsten Wissel, Gudrun Brandes, Nils Pütz, Gian Luigi Angrisani, Jan Thieleke, Thomas Lenarz, Martin Durisin

**Affiliations:** 1 Department of Otorhinolaryngology, Hannover Medical School, Hannover, Germany; 2 Cluster of Excellence ‘Hearing 4 all’, NIFE, Hannover, Germany; 3 Institute of Neuroanatomy and Cell Biology, Center of Anatomy and Cell Biology, Hannover Medical School, Hannover, Germany; 4 Biotechnology Center, TU Dresden, Dresden, Germany; 5 Institute of Materials Science, Leibniz University Hannover, Garbsen, Germany; 6 Institute of Inorganic Chemistry, Leibniz University Hannover, Hannover, Germany; Hungarian Academy of Sciences, HUNGARY

## Abstract

Despite the technological progress made with cochlear implants (CI), impedances and their diagnosis remain a focus of interest. Increases in impedance have been related to technical defects of the electrode as well as inflammatory and/or fibrosis along the electrode. Recent studies have demonstrated highly increased impedances as the result of corroded platinum (Pt) electrode contacts. This *in vitro* study examined the effects of Pt ions and compounds generated by corrosion of the electrode contacts of a human CI on cell metabolism. Since traces of solid Pt in surrounding cochlear tissues have been reported, the impact of commercially available Pt nanoparticles (Pt-NP, size 3 nm) on the cell culture model was also determined. For this purpose, the electrode contacts were electrically stimulated in a 0.5% aqueous NaCl solution for four weeks and the mass fraction of the platinum dissolute (Pt-Diss) was determined by mass spectrometry (ICP-MS). Metabolic activity of the murine fibroblasts (NIH 3T3) and the human neuroblastoma (SH-SY5Y) cells was determined using the WST-1 assay following exposure to Pt-Diss and Pt-NP. It was found that 5–50 μg/ml of the Pt-NP did not affect the viability of both cell types. In contrast, 100 μg/ml of the nanoparticles caused significant loss in metabolic activity. Furthermore, transmission electron microscopy (TEM) revealed mitochondrial swelling in both cell types indicating cytotoxicity. Additionally, TEM demonstrated internalized Pt-NP in NIH 3T3 cells in a concentration dependent manner, whereas endocytosis in SH-SY5Y cells was virtually absent. In comparison with the Pt-NP, the corrosion products (Pt-Diss) with concentrations between 1.64 μg/ml and 8.2 μg/ml induced cell death in both cell lines in a concentration dependent manner. TEM imaging revealed both mitochondrial disintegration and swelling of the endoplasmic reticulum, suggesting that Pt ions trigger cytotoxicity in both NIH 3T3 and SH-SY5Y cell lines by interacting with the respiratory chain.

## Introduction

Cochlear implantation (CI) represents the only therapeutic intervention for patients with profound sensory neural hearing loss to date [1–2]. However, insertion of the CI into the scala tympani induces trauma in turn resulting in inflammation processes, fibrosis on the implant surface and new bone formation inside the scala tympani [3–8]. It was shown that those foreign-body reactions result in an increase of electrode impedance and consequently in a decrease of the electrical dynamic range for stimulation. Thus, greater electrical stimuli are required for sufficient neuronal stimulation leading to higher energy consumption [9–12]. In cases of persistent high-impedance values neural response telemetry and reprogramming of the implant in conjunction with administration of cortisone and antibiotics usually lead to normalization of the impedance values. Recently, several cases of recurrent increases in impedance in the same channels have been found which could not be explained by inflammation processes alone [13–14]. Investigation of explanted electrodes by scanning electron microscopy (SEM) revealed not only the presence of connective tissue with traces of blood, but also corrosion of the platinum surface of the electrode contacts [8, 14–15]. Furthermore, SEM micrographs of platinum microelectrodes demonstrated corrosion of Pt following stimulation in various biological buffer solutions [16].

Due to its electrochemical stability and biocompatibility Pt has been preferentially used as electrode material for neuroprostheses for some decades now. There are, however, electrochemical processes at the electrode-electrolyte interface involving corrosion of noble metals: Intrinsic charge injection is limited for every electrode and, therefore, the voltage that can be safely generated at the electrode surface is restricted. In the event that this voltage is exceeded, it is not possible to maintain purely capacitive charge transfer and, subsequently, irreversible faradaic processes occur. Those reactions comprise both cathodic and anodic water hydrolysis, the oxidation of chloride ions, release of oxygen gas and the oxidation of platinum [17–20].

Early studies on Pt dissolution suggested that Pt^2+^ ions and their complexes with chloride oxidation products as like as ClO^-^ and ClO^3-^ are the most likely dissolved species at the Pt/saline interface [17, 21]. Additionally, time of flight secondary ion mass spectrometry (TOF-SIMS) analysis of the tissue-platinum electrode interface following CI explantation identified not only oxidized PtO^-^ and PtO^2-^, but also associates of Pt with sodium and calcium ions, Pt-protein-complexes and carbohydrates [8]. In previous studies, it was found that the presence of Pt-protein-complexes significantly reduced the extent of Pt dissolution. They were, by physical adsorption, able to form protective films which restrict the diffusion of reactants and products to and from the metal surface [22–24]. Furthermore, both platinum oxidation and oxide reduction were blocked following absorption of certain amino acids on the platinum surface. It has been suggested that several amino acids compete with chloride derived from phosphate-buffered saline [24]. However, amino acids with sulfur-hydrogen groups such as cysteine were known to interact strongly with platinum increasing the corrosion in a concentration-dependent manner [24].

Regarding the potential cytotoxicity of Pt ions and complexes, there is evidence of long-term toxic effects on bacteria, eukaryotic cells, and both mammalian and non-mammalian tissues [25–29]. In particular, cis-platinum compounds, e.g. cis-diamminedichloroplatinum(II) (cis-[Pt(NH_3_)_2_Cl_2_]), are used as potent anti-cancer drugs [26, 30–31].

In addition to Pt ions and its complexes traces of solid Pt particles were also found in the spiral ligament, connective tissue around the electrode, macrophages and even in the middle ear [8, 14, 32–34]. *In vitro* cytotoxic and genotoxic effects of Pt nanoparticles with average size below 10 nm have not been demonstrated at concentrations below 80–100 μg/ml in several cell lines thus far [35–38]. However, it has also been reported that–depending on the cell type and organism–Pt-NP induced oxidative stress, DNA damage, inflammation or other negative biological effects in a concentration-depending manner [39–44].

Therefore, it is of great clinical interest that we comprehend the process involved in formation of Pt corrosion products at the electrode-tissue interface, their distribution within the cochlea and their interaction with the neuronal cells. The present study examined the effects of Pt particles and compounds on an *in vitro* cell culture model. This involved electrically stimulating the electrode contacts of a human CI electrode for four weeks in an aqueous 0.5% NaCl solution. The Pt-containing dissolute (Pt-Diss) was administered to both the murine fibroblast cell line NIH 3T3 and the human neuroblastoma cell line SH-SY5Y in varying concentrations. Since previous studies reported traces of solid Pt within cochlear structures [8, 14, 32–34], commercially available Pt nanoparticles (Pt-NP, size 3 nm) were included in this study. The potential cytotoxicity of both Pt-Diss and Pt-NP in a concentration-dependent manner was examined using the WST-1 assay, and the ultrastructural changes were documented by transmission electron microscopy (TEM).

## Materials and methods

### Electrical stimulation of the human CI electrode

The cochlear implant electrode used for this study was constructed in accordance with the design of the RE24 Contour Advance™ electrode for research purposes (Cochlear Ltd., Macquarie University, NSW, Australia). Electrical stimulation was performed in an electrolyte consisting of 0.5% NaCl (Braun, Melsungen, Germany) in deionized water for 600 h at room temperature; it was carried out. using a function generator (Agilent Technologies, Santa Clara, CA, USA) and amplified using a linear amplifier (Brüel & Kjær, Nærum, Denmark). The stimulation signal was a biphasic square wave with a duty cycle of 50% and a frequency of 5,000 Hertz resulting in alternating impulses of 100 μs with no interpulse delay. The voltage amplitude was 20.0 volts with a current of 0.3 amperes.

### Determination of the platinum (Pt) content following electrical stimulation

The total amount of platinum (Pt) in the 0.5% NaCl solution was analysed by inductive coupled plasma-mass spectrometry (ICP-MS). The sample solution was diluted 1:10 with Millipore® high purity water and ultrapure nitric acid (p.a., Merck KGaA, Darmstadt, Germany), further purified by sub-boiling distillation to achieve a matrix matching of standards and samples. The standards were prepared by using ICP platinum standards (Carl Roth GmbH, Karlsruhe, Germany) and diluted ultrapure nitric acid (ω = 2.5%). Quantification was performed with external calibration by ICP-MS (X-Series 2, Thermo Fisher Scientific Inc., Waltham, USA). Evaluation and validation of the analysis was carried out in accordance with relevant DIN standards [45–46].

### Dispersion of platinum nanoparticles (Pt-NP)

A 20 mg/ml stock solution of hydrophilic Pt-NP powder (average particle size: 3 nm), stabilized with polyvinylpyrrolidone (PlasmaChem, Berlin, Germany) was prepared by dispersion in sterile aqua bidest., followed by sonication in a water bath for 15 min.

### Seeding and cultivation of NIH 3T3 and SH-SY5Y cells followed by supplementation of Pt nanoparticles (Pt-NP) and dissolutes (Pt-Diss)

Both the murine cell line NIH3T3 and the human neuroblastoma cell line SH-SY5Y (Leibniz Institute DSMZ—German Collection of Microorganisms and Cell Cultures GmbH, Braunschweig, Germany) were seeded into 96-well microtiter plates (Nunclon, Thermo Fisher Scientific, Kempen, Germany) at a density of 2 x 10^3^ NIH 3T3 and 5 x 10^3^ SH-SY5Y cells per well. Cultivation was performed in 100 μl high glucose DMEM containing 10% fetal bovine serum (FBS) (all components by Biochrom AG, Berlin, Germany) at 37°C and 5% CO_2_ for 24 h. The culture medium was removed and replaced by 100 μl of the Pt-containing solutions at their final concentrations (Pt-Diss, pH 5.0: 0.82–8.2 μg/ml in culture medium, pH 7.5–8.0, Pt-NP: 5–100 μg/ml in culture medium, pH 7.5–8.0). Cell cultivation was continued for another 48 h (NIH 3T3 cells) and 6 d (SH-SY5Y cells).

As described above, NaCl was used as the electrolyte for electrical stimulation and, therefore, changes in the NaCl concentration of the culture medium have to be considered due to supplementation of the cell culture assays with varying Pt-Diss concentrations. In order to exclude adverse effects of NaCl on cell metabolism, cultivation assays containing medium with final NaCl concentrations of 6.26, 6.12 and 5.93 mg/ml were included in this study. For this purpose NaCl (Merck GmbH, Darmstadt, Germany) was dissolved in bi-distilled water to a stock concentration of 5 mg/ml and sterile filtrated. Cultivation assays for both cell lines were set up as described above, and cultivated for 24 h prior to incubation in culture medium supplemented with the NaCl solution.

Since the completed DMEM culture medium also contains NaCl at a concentration of 6.4 mg/ml, dilution with 1:10, 1:5 and 1:3 NaCl stock corresponded to the final NaCl concentration of 6.26, 6.12 and 5.93 mg/ml respectively.

All cultivation assays were analysed using light microscopy (Zeiss Axio Observer Z1, Zeiss, Jena, Germany) and the images were captured digitally by a CCD colour camera (Hitachi HV-D30, Hitachi Kokusai Electric, Japan).

### Determination of the effects of Pt-NP and Pt-Diss on the metabolic activity of NIH 3T3 and SH-SY5Y cells using the WST-1 assay

The metabolic activity of both cell types was determined by the conversion of the tetrazolium compound WST-1 to a formazan dye as the result of the reduction of WST-1 by mitochondrial dehydrogenases [47]. It was found that an increase in the optical density (OD) of the formazan dye directly correlates with the metabolic activity of the cells following exposure to either Pt-Diss or Pt-NP. For measurement of the formazan dye intensities, the cell culture medium was exchanged with those containing WST-1 solution at the final concentration of 1 mg/ml. Depending on cell type, the samples were incubated at 37°C for 45 min (NHI 3T3 cells) and 2 h (SH-SY5Y cells). The absorption of the formazan dye was measured at λ = 450 nm and at λ = 650 nm (the reference wave length) using the microplate reader (SynergyH1, Biotek, Bad Friedrichshall, Germany). Cells without any treatment were used as a reference, and wells containing culture medium alone served as a background control. To determine the relative metabolic activity of the culture assays supplemented with either Pt-Diss or Pt-NP, the resulting ODs were related to those obtained from the reference and calculated as a percentage [%]. In accordance with ISO 10993–5 [48], cell viability lower than 70% (in comparison with the reference) was deemed cytotoxic.

Each assay was performed at least in triplicate in n = 3 independent experiments.

### Scanning electron microscopy (SEM)

The Pt electrode contacts of the human cochlear implant that underwent electrical stimulation were examined using SEM (Supra 55 VP, Carl Zeiss GmbH, Jena, Germany). Using the BSE detector, images were generated at a pressure of 6.64 x 10^−6^ mbar, a working distance of 11.5 mm and a tube voltage of 15 kV.

### Transmission electron microscopy (TEM)

Both cell lines were seeded into a 6-well microtiter plate (Nunclon, Thermo Fisher Scientific, Kempen, Germany) at densities of 3.5 x 10^5^ cells per well and cultivated in 3 ml supplemented high glucose DMEM for 24 h as described above. In order to investigate the effects of Pt–as either nanoparticles or ionic components–on the ultrastructure both cell lines were cultivated in 5 ml culture medium containing either 5–100 μg/ml of Pt-NP or 0.02–6 μg/ml of the Pt-Diss for another 48 h and 6 d, respectively. Additionally, untreated cells–both in the medium alone and in culture medium supplemented with the same NaCl concentration as present in the Pt-Diss solution–were included as controls. The cells were collected using a cell scraper and centrifuged at 1000 rpm for 4 min (Micro Star 17, VWR, Radnor, PA, USA) and fixed with 2.5% glutardialdehyde (Polysciences, Warrington, PA, USA) in 0.1 M sodium cacodylate (Th. Geyer, Hamburg, Germany). After post-fixation with 2% osmium tetroxide (Polysciences) in 0.1 M sodium cacodylate the cell pellets were embedded in epoxide resin (Serva, Heidelberg, Germany). Ultra-thin sections stained with 2% uranyl acetate (Serva) and lead citrate (Serva) were examined under the transmission electron microscope (Tecnai G2 200 kV TEM, Eindhoven, Netherlands). The digital images were processed using Adobe Photoshop CS6.

### Statistical analysis

All data achieved from the in vitro cell culture assays were presented as mean ± standard error of mean (SE_M_). One-way nonparametric analysis of variance (ANOVA) and Newman-Keuls multiple comparison tests were used for statistical assessment.

## Results

### Determination of the effects of additional NaCl in the cell cultivation assay

**Fig 1:**
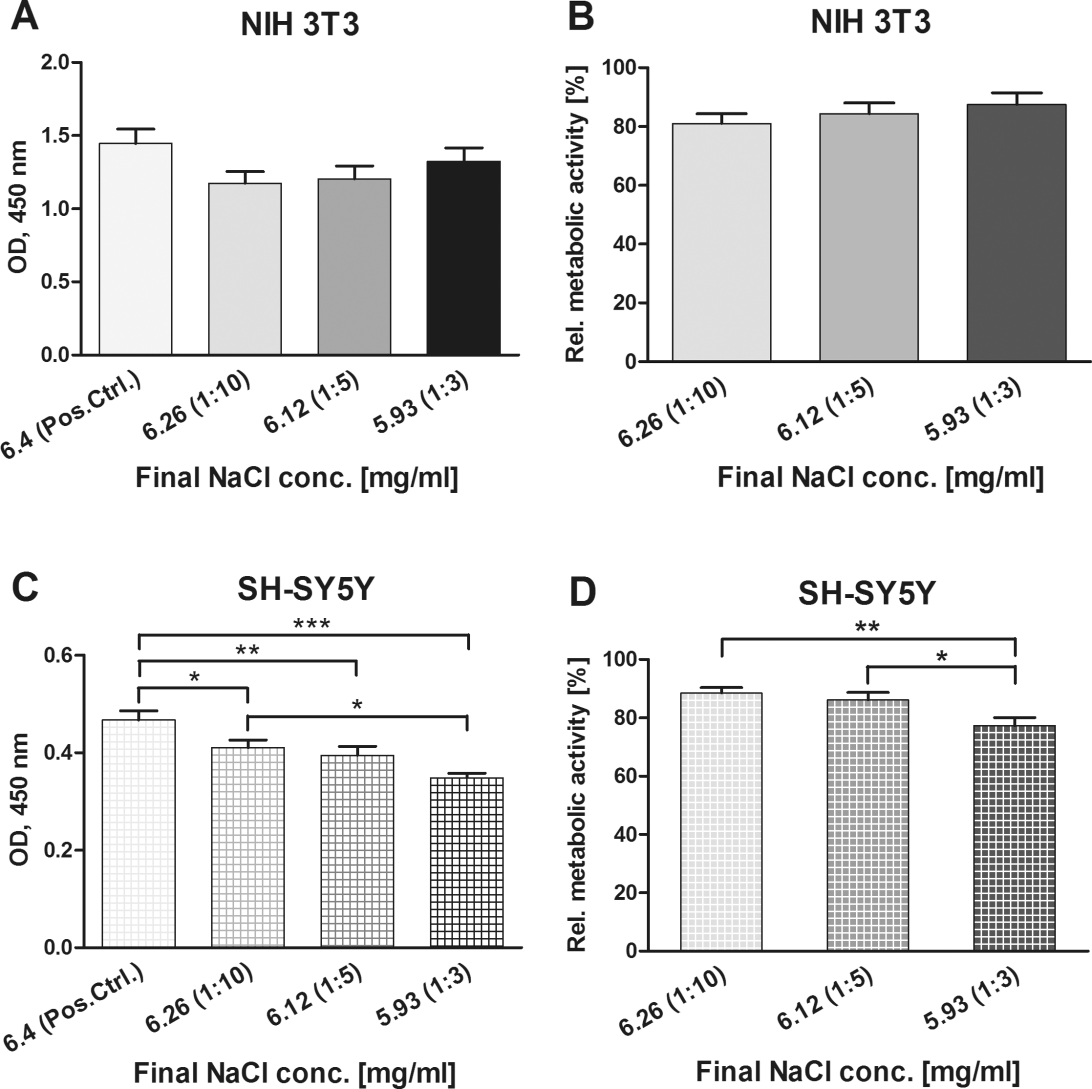
Determination of the effects of additional NaCl input in cell cultivation. Metabolic activity of both NIH 3T3 (**A**, **B**) and SH-SY5Y cells (**C**, **D**) grown in culture medium containing 6.4 (Pos.Ctrl., reference), 6.26, 6.12 and 5.93 mg/ml NaCl corresponding to 1:10, 1:5 and 1:3 dilution of the NaCl stock in culture medium was determined by indirect reduction of WST-1 by mitochondrial dehydrogenases to a formazan dye. Optical densities (OD) were measured in 48 h and 6 d cultivation assays (NIH 3T3, n = 13–23; SH-SY5Y, n = 24–27). The resulting formazan dye intensities were also related to those obtained from the reference and calculated as a percentage [%]. Each data point is presented as mean and SE_M_. ANOVA with Newman-Keuls multiple comparison test was performed for statistical assessment (***p ≤ 0.001, *p ≤ 0.05).

To exclude adverse effects of NaCl on cell growth and functionality cell culture assays containing medium with final NaCl concentrations of 6.26, 6.12 and 5.93 mg/ml were included in the investigation of potential cytotoxicity of Pt-Diss. In comparison to the reference with 6.4 mg/ml NaCl in the completed DMEM culture medium (OD 1.445 ± 0.01, standard cultivation assay), the metabolic activity of the NIH 3T3 cells did not significantly change in the different mixtures of 0.5% NaCl and DMEM medium (Fig 1A): The OD of the fibroblast cultivation assays with final NaCl concentrations of 6.26, 6.12 and 5.93 mg/ml varied between 1.175 ± 0.079 (NaCl 6.26 mg/ml) and 1.324 ± 0.092 (NaCl 5.93 mg/ml). Additionally, microscopic control revealed stable attachment and normal morphology of the NIH 3T3 cells independently of NaCl supplementation. As shown in Fig 1B, no significant impairment of cell metabolism could be found in cultivation assays with varying NaCl concentrations: Metabolic activity in relation to the reference ranged between 81.04% ± 3.31 (NaCl 6.26 mg/ml) and 87.5% ± 3.95 (NaCl 5.93 mg/ml).

**Fig 2:**
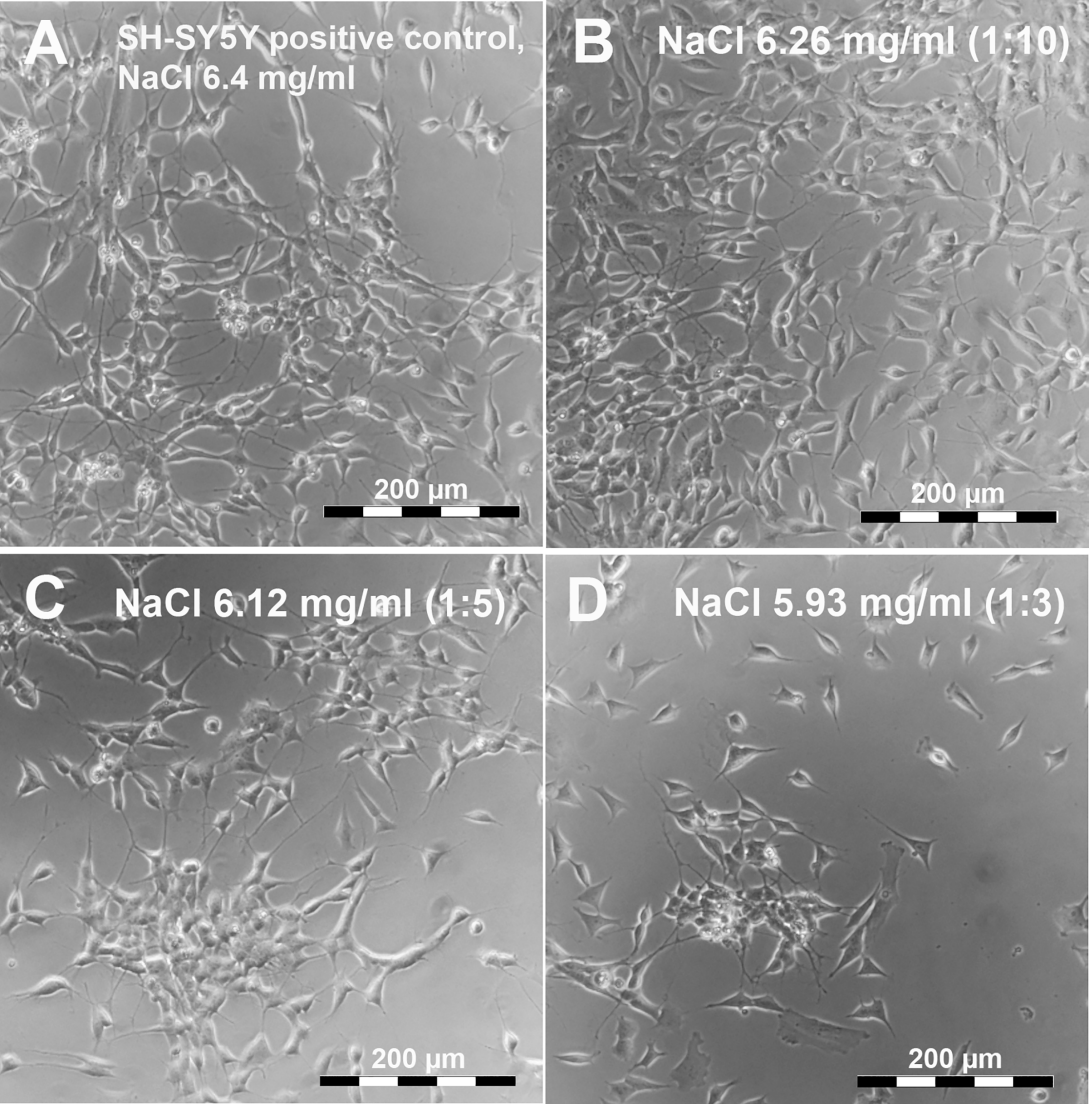
Microscopic characterization of the morphology of SH-SY5Y cells following exposure to varying NaCl concentrations. SH-SY5Y cells were cultivated either in normal cell culture medium containing 6.4 mg/ml NaCl as reference (**A**) or in culture medium containing 6.26 mg/ml (1:10) (**B**), 6.12 mg/ml (1:5) (**C**) and 5.93 mg/ml (1:3) NaCl (**D**). Microscopic images demonstrated reduced cell attachment and growth in a concentration dependent manner without any signs of cytotoxicity.

As presented in Fig 1C, the neuroblastoma cell line SH-SY5Y demonstrated a significant decrease in metabolic activity at final NaCl concentrations of 5.93 mg/ml (OD 0.348 ± 0.010) and 6.12 mg/ml (OD 0.394 ± 0.019) in comparison with the reference (OD 0.467 ± 0.019). By contrast, 6.26 mg/ml NaCl in the medium resulted in a slight, but distinct, decline in metabolic activity (OD 0.410 ± 0.016) compared with the reference. Relative to the cell metabolism of the reference, however, cell viability in culture medium containing 6.26, 6.12 and 5.93 mg/ml was diminished to 88.50% (± 1.88), 86.17% (± 2.57) and 77.37% (± 2.76) respectively, presenting a significant difference in relative metabolic activity between 6.26 mg/ml and 5.93 mg/ml NaCl content. According to these findings, none of the cultivation assays with differing NaCl concentrations resulted in a decrease in cell viability below 70% relative to the reference indicating any cytotoxic effects. By contrast, microscopic imaging revealed stable adhesion and intact morphology of the SH-SY5Y cells in medium containing 6.26 mg/ml, 6.12 mg/ml and 5.93 mg/ml NaCl. Only the growth rate seemed to be retarded (Fig 2B–2D). We concluded that dilution of the culture medium with NaCl supplemention may limit the SH-SY5Y cell growth (as indicated by a decrease in overall metabolic activity), but did not impair cell differentiation.

### SEM view of eroded Pt electrode contacts of a human CI

**Fig 3:**
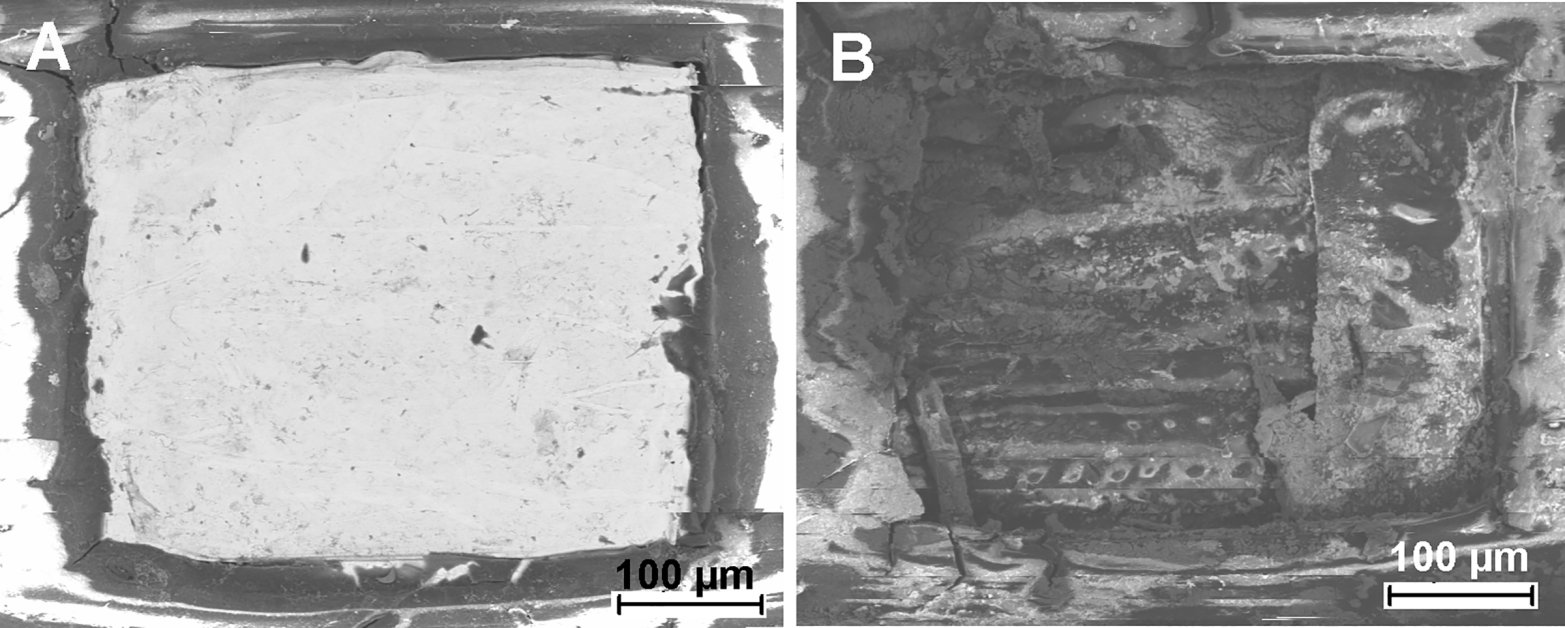
SEM characterization of eroded Pt electrode contacts of a human CI. Representative BSE-image of the CI-array used for electrical stimulation for 600 h at room temperature in an electrolyte consisting of 0.5% NaCl in deionized water. (**A**) represents an intact Pt-electrode contact, whereas corrosion of the Pt-electrode contact following electrical stimulation could be demonstrated (**B**).

Following electrical stimulation of the human CI the surface structure of the electrode contacts were examined by SEM. Fig 3 shows the electrode contacts before and after electrical stimulation for 600 h. While the Pt contact had a glossy surface without any irregularities prior to electrical stimulation (Fig 3A), strong corrosion was demonstrated as the result of induction of faradaic processes (Fig 3B).

### Pt-Diss showed strong cytotoxic effects in both cell lines

After electrical stimulation of the Pt-electrode contacts the Pt-Diss was added to the NIH 3T3 and SH-SY5Y cell cultures in varying concentrations.

**Fig 4:**
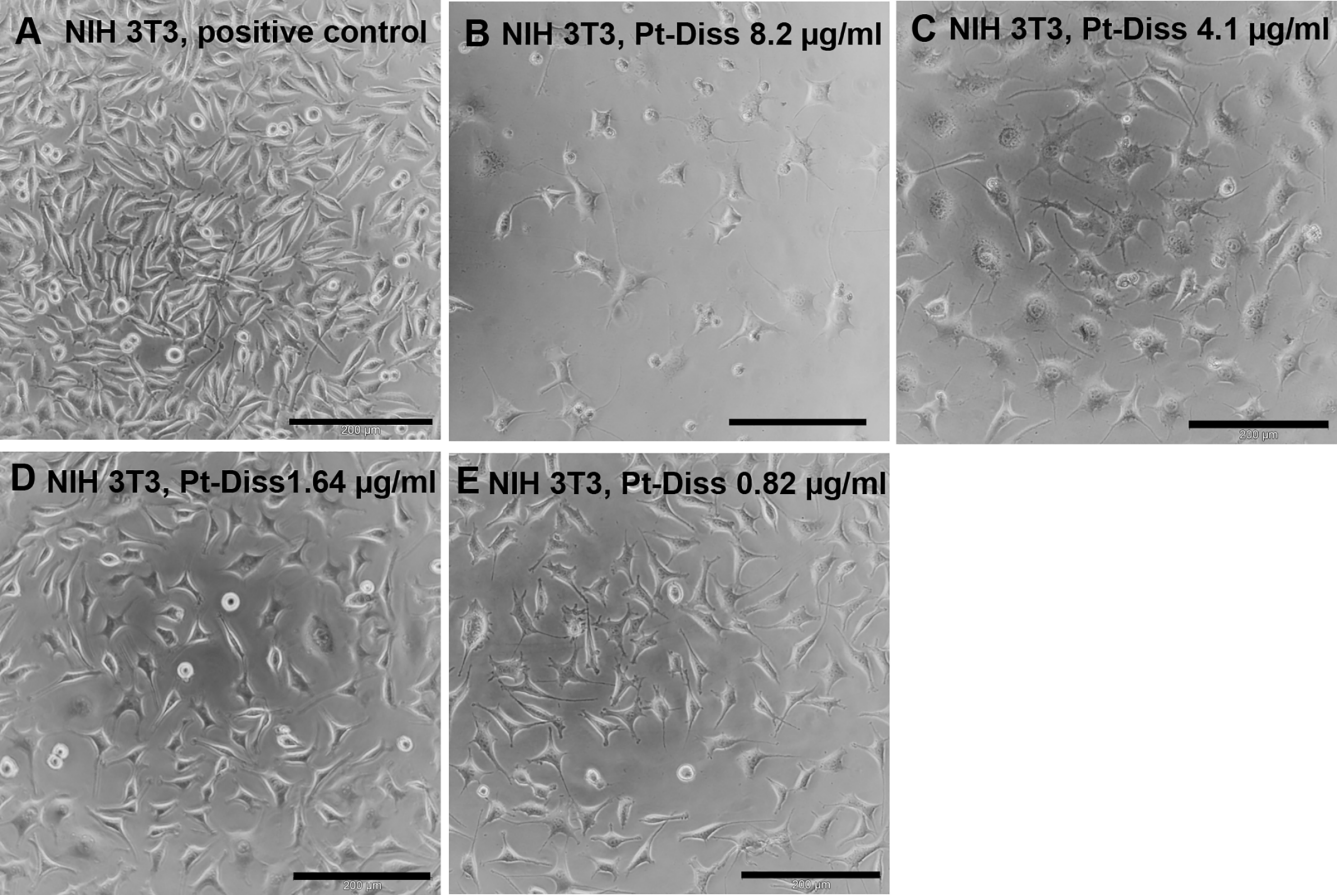
Microscopic characterization of the morphology of NIH 3T3 cells following exposure to Pt-Diss. NIH 3T3 cells were cultivated either without any additional Pt-Diss as reference (**A**) or in culture medium containing 8.2 μg/ml (**B**), 4.1 μg/ml (**C**), 1.64 μg/ml (**D**) and 0.82 μg/ml (**E**) of the Pt components. Microscopic images demonstrated emerging cytotoxic effects of Pt-Diss in a concentration-dependent manner between 1.64 μg/ml and 8.2 μg/ml Pt-Diss concentration. Beyond Pt-Diss concentration of 1.64 μg/ml cell adhesion and morphology appeared normal, whereas a Pt-Diss concentration of 8.2 μg/ml strongly reduced NIH 3T3 cell growth and probably induced cell death. Size of bars: 200 μm.

The microscopic view of NIH 3T3 fibroblasts showed–in comparison with the reference (cultivation assay without additional NaCl and Pt)–a strong reduction in cell growth (Fig 4A and 4B). Additionally, shrunk cell bodies probably undergoing cell death were observed at the Pt-Diss concentration of 8.2 μg/ml Pt (Fig 4B). Adhesion and growth were inversely correlated to Pt-Diss concentration (Fig 4). Beyond a concentration of 1.64 μg/ml Pt cell adhesion and morphology appeared normal (Fig 4D).

**Fig 5:**
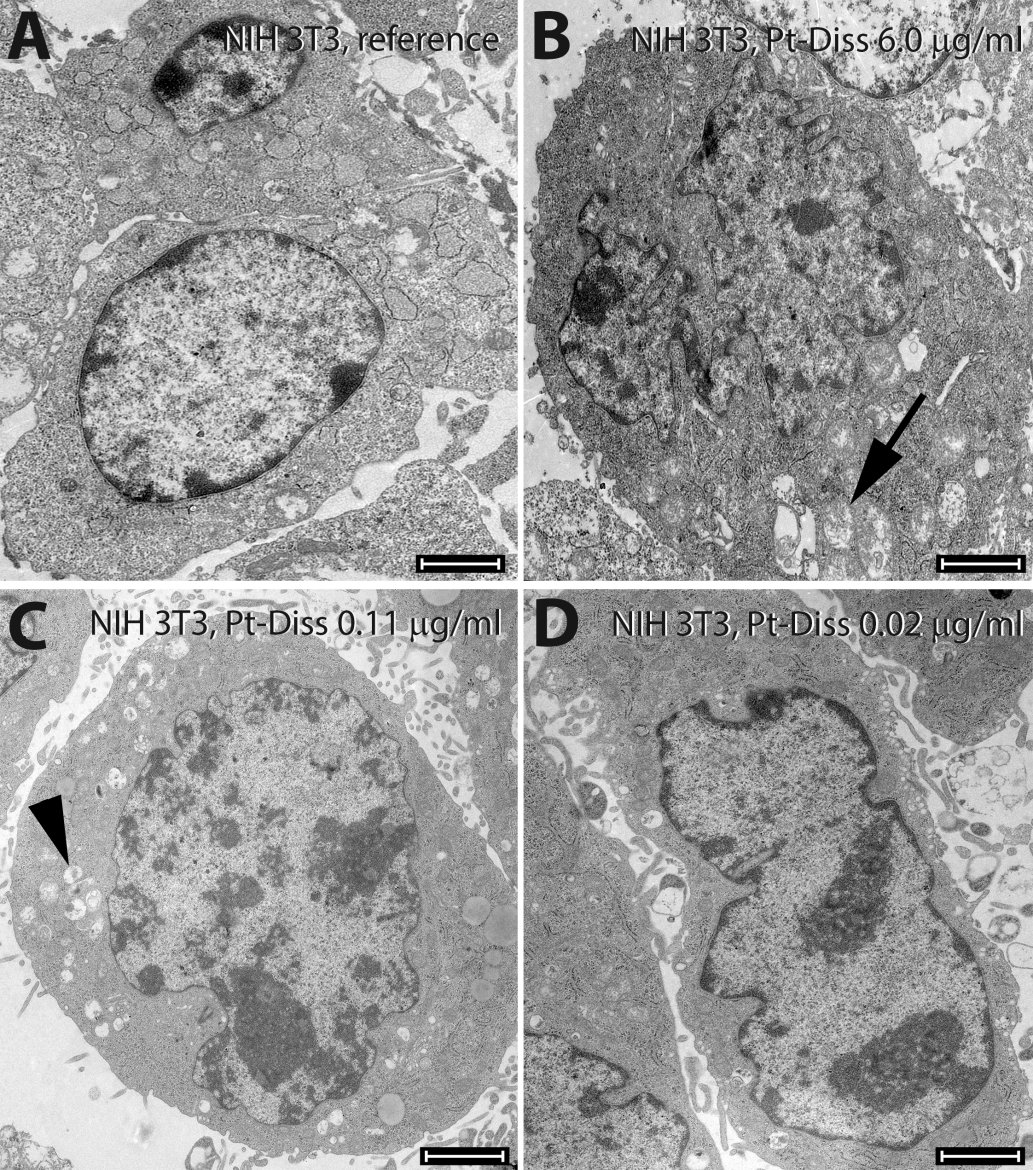
Ultrastructural morphology of NIH 3T3 cells following exposure to Pt-Diss. After cultivation either without Pt as reference (**A**) or in culture medium containing 6.0 μg/ml (**B**), 0.11 μg/ml (**C**) or 0.02 μg/ml Pt (**D**) the ultrastructure of 3T3 fibroblasts was compared. They demonstrated mitochondrial swelling (arrow in **B**) only at the highest tested Pt concentration. At 0.11 μg/ml Pt the cells showed greater phagocytic activity (arrowhead in **C**). Lower amount of Pt in the culture medium induced no morphological changes in comparison with the control. Size of bars: 2 μm.

Our observations were confirmed by TEM imaging that revealed induction of cytotoxic signals, especially in the mitochondria of fibroblasts at Pt-Diss concentrations of 6 μg/ml Pt (Fig 5B). The cells had pronounced phagocytic activity at Pt-Diss of 0.11 μg/ml Pt (Fig 5C). However, cells exposed to 0.02 μg/ml Pt could not be distinguished from those cultivated in medium alone (reference) as or in NaCl-diluted culture mixtures (Fig 5A and 5D).

**Fig 6:**
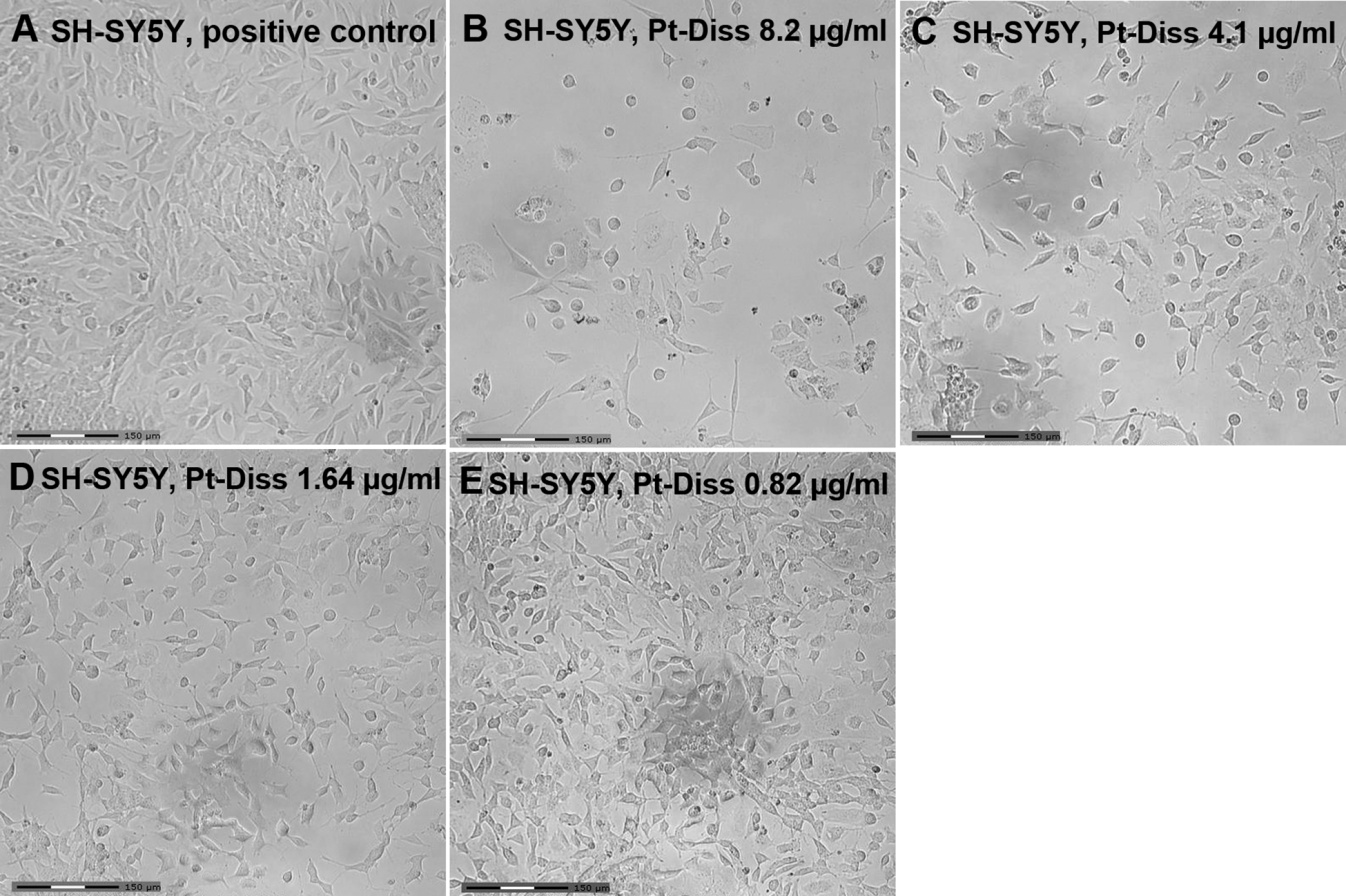
Microscopic characterization of the morphology of SH-SY5Y cells following exposure to Pt-Diss. SH-SY5Y cells were cultivated either without any additional Pt-Diss as reference (**A**) or in culture medium containing 8.2 μg/ml (**B**), 4.1 μg/ml (**C**), 1.64 μg/ml (**D**) and 0.82 μg/ml (**E**) of the Pt components. Microscopic images demonstrated emerging cytotoxic effects of Pt in a concentration dependent-manner between 1.64 μg/ml and 8.2 μg/ml Pt-Diss concentration. Cell adhesion and growth appeared stable following exposure to Pt-Diss concentration of around 1.64 μg/ml. Even spontanous neurite sprouting could be observed, whereas the 8.2 μg/ml of the Pt-Diss concentration strongly induced detachment of the SH-SY5Y cells and subsequent cell death. Size of bars: 150 μm.

The neuroblastoma cell line SH-SY5Y demonstrated (in comparison with the reference) a growth reduction over 6 d depending on the concentration of Pt-Diss in the medium (Fig 6). In a similar manner to observations of the NIH 3T3 cells after Pt-Diss exposure, few SH-SY5Y cells attached to the tissue culture plate following application of the Pt-Diss concentration of 8.2 μg/ml Pt. The somata partially exhibited spherical morphology indicating induction of apoptosis (Fig 6B). Neuroblastoma cells cultivated with 0.82 μg/ml Pt indicated normal cell adhesion and morphology–even spontaneous neurite sprouting could be observed (Fig 6A and 6E).

**Fig 7:**
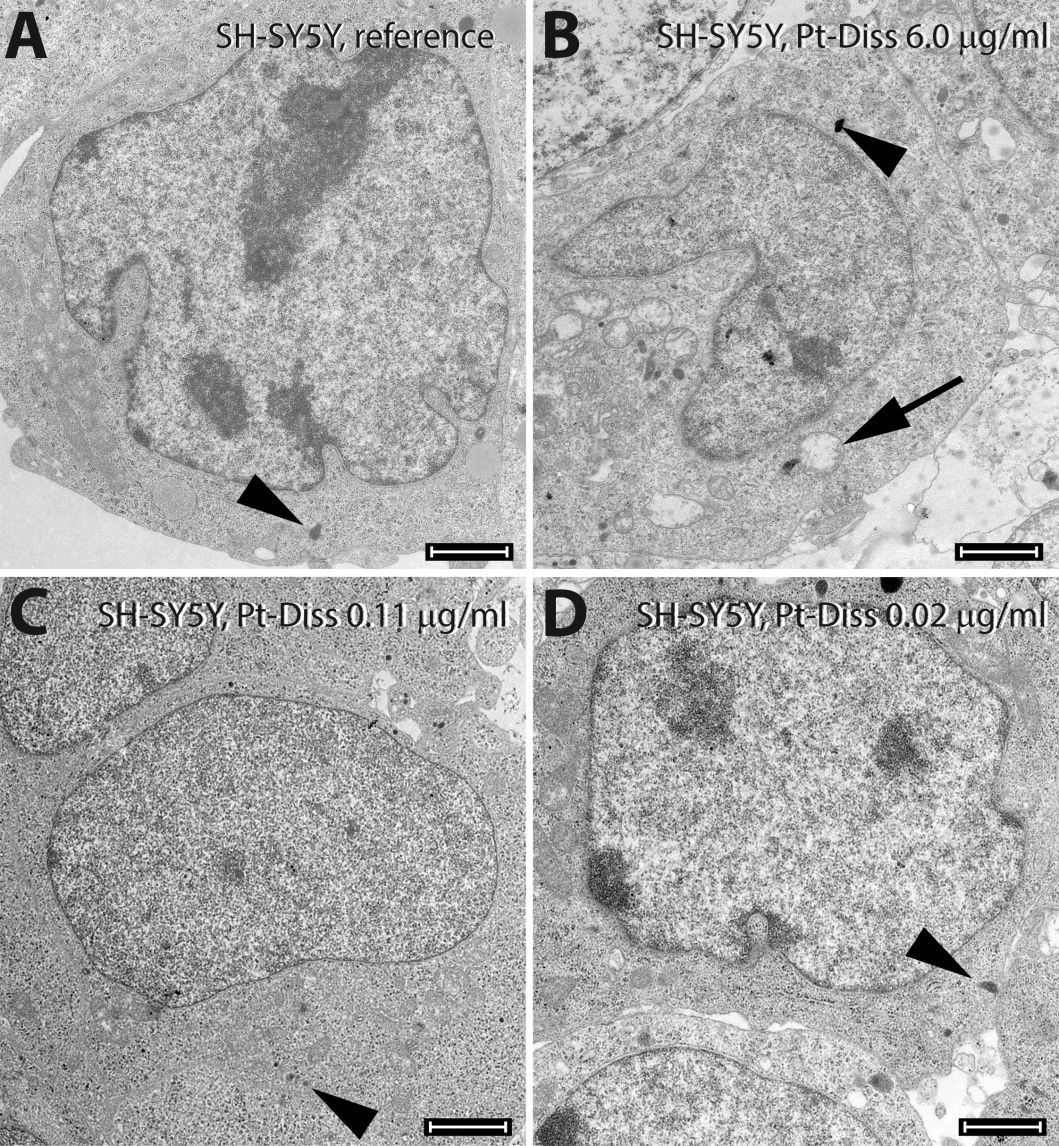
Ultrastructure of the SH-SY5Y cell line cultivated in absence and presence of Pt-Diss. After cultivation either without any Pt as reference (**A**) or in culture medium containing 6.0 μg/ml (**B**), 0.11 μg/ml (**C**) or 0.02 μg/ml Pt (**D**) SH-SY5Y were characterized by large euchromatic nucleus, abundant endoplasmic reticulum and a few synaptic granules (arrowheads). At the highest tested Pt concentration these cells were adversely affected, as proven by mitochondrial swelling (arrow in **B**). A smaller amount of Pt in the culture medium induced no morphological changes in comparison with the control. Size of bars: 2 μm.

The ultrastructure of the neuroblastoma cells confirmed cytotoxic signals, especially in the mitochondria, but only at Pt-Diss concentrations of 6 μg/ml Pt (Fig 7B). As also described for the NIH 3T3 cells, the morphology of the SH-SY5Y cells following incubation in culture medium supplemented with 0.02 μg/ml or 0.11 μg/ml Pt was similar in to the cells of the reference and of the corresponding NaCl control. As shown in Fig 7A, 7C and 7D, cells exhibited euchromatic nuclei, abundant endoplasmic reticulum and electron-dense synaptic granules. Additionally, no phagocytic activity could be observed (Fig 7A, 7C and 7D).

**Fig 8:**
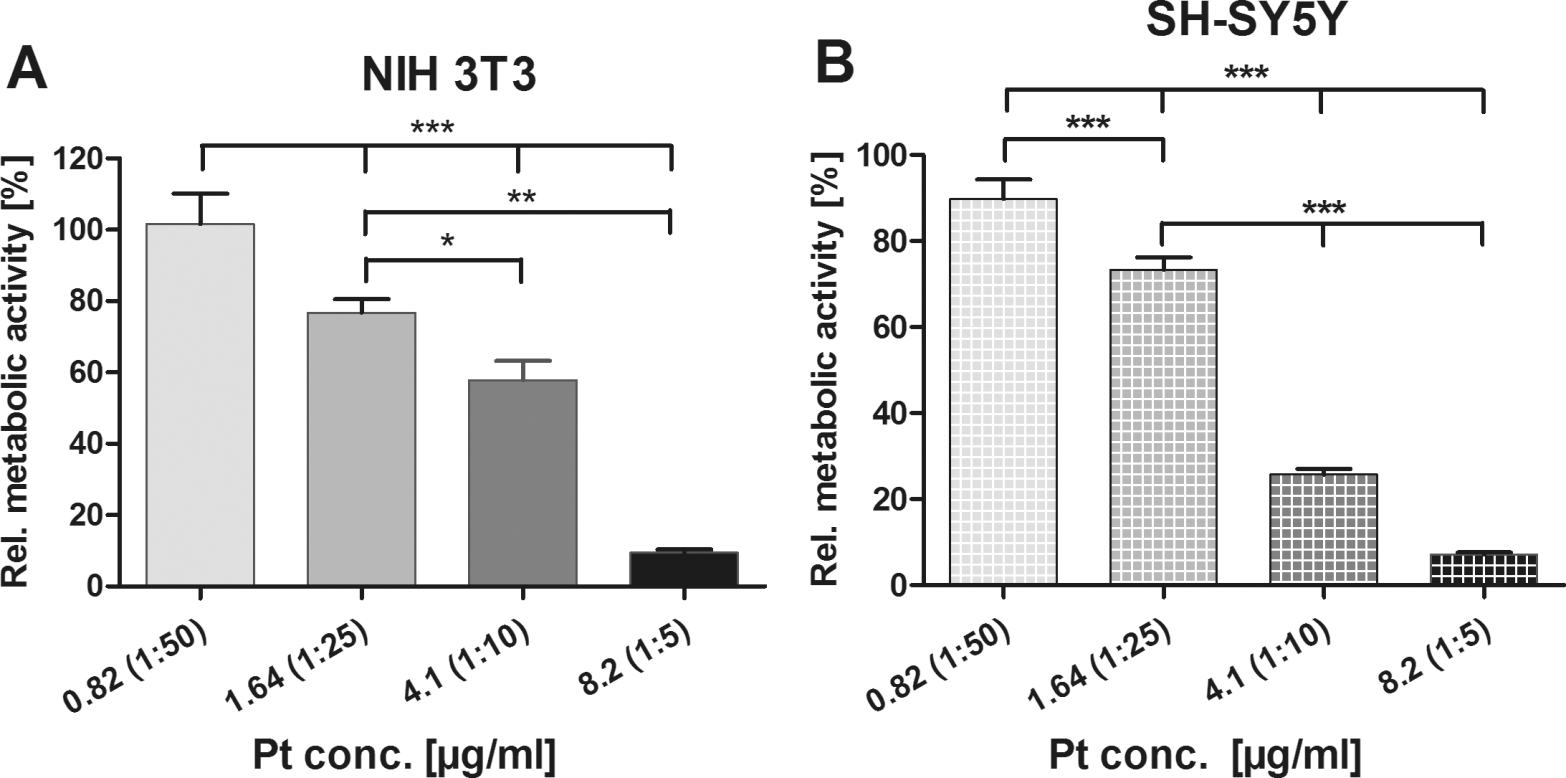
Determination of the effects of Pt-Diss with varying Pt-Diss concentrations in cell cultivation. Metabolic activity of both NIH 3T3 (**A**) and SH-SY5Y cells (**B**) grown in culture medium supplied with 0.82 μg/ml– 8.2 μg/ml Pt-Diss concentration was determined by indirect reduction of WST-1 by mitochondrial dehydrogenases to a formazan dye. Optical densities (OD) were measured in 48 h and 6 d cultivation assays (NIH 3T3, n = 12–16; SH-SY5Y, n = 10). The resulting formazan dye intensities were related to those obtained from the reference and calculated as a percentage [%]. Each data point is presented as mean and SE_M_. ANOVA with Newman-Keuls multiple comparison test was performed for statistical assessment (***p ≤ 0.001, **p ≤ 0.01, *p ≤ 0.05).

Cell viability following 48 h and 6 d cultivation was determined using the WST-1 assay. The optical densities of the samples resulting from the indirect reduction of the tetrazolium salt to the formazan dye were related to the reference. In accordance with the microscopic characterization, we found that metabolic activity decreased in a concentration-dependent manner (Fig 8). Highly significant downregulation of metabolic activity was found in both cell types (NIH 3T3: 9.44% ± 0.93; SH-SY5Y: 7.10% ± 0.51) following administration of 8.2 μg/ml Pt-Diss (Fig 8). However, at the Pt-Diss concentration of 4.1 μg/ml metabolism of the neuroblastoma cells seemed to be more affected (25.70% ± 1.32) in comparison with the fibroblasts (57.86% ± 5.39). The administration of Pt-Diss at a concentration of 1.64 μg/ml Pt resulted in significantly improved cell viability to 76.70% ± 1.32 and 73.32% ± 2.89 in in NIH 3T3 and SH-SY5Y cells, respectively. Finally, the tenfold lower Pt-Diss concentration of 0.82 μg/ml did not impair the metabolic activity in either of the cell lines (NIH 3T3 101.60% ± 8.55; SH-SY5Y 89.77% ± 4.52).

### Pt-NP of up to 50 μg/ml did not cause cell damage in NIH 3T3 and SH-SY5Y cells

**Fig 9:**
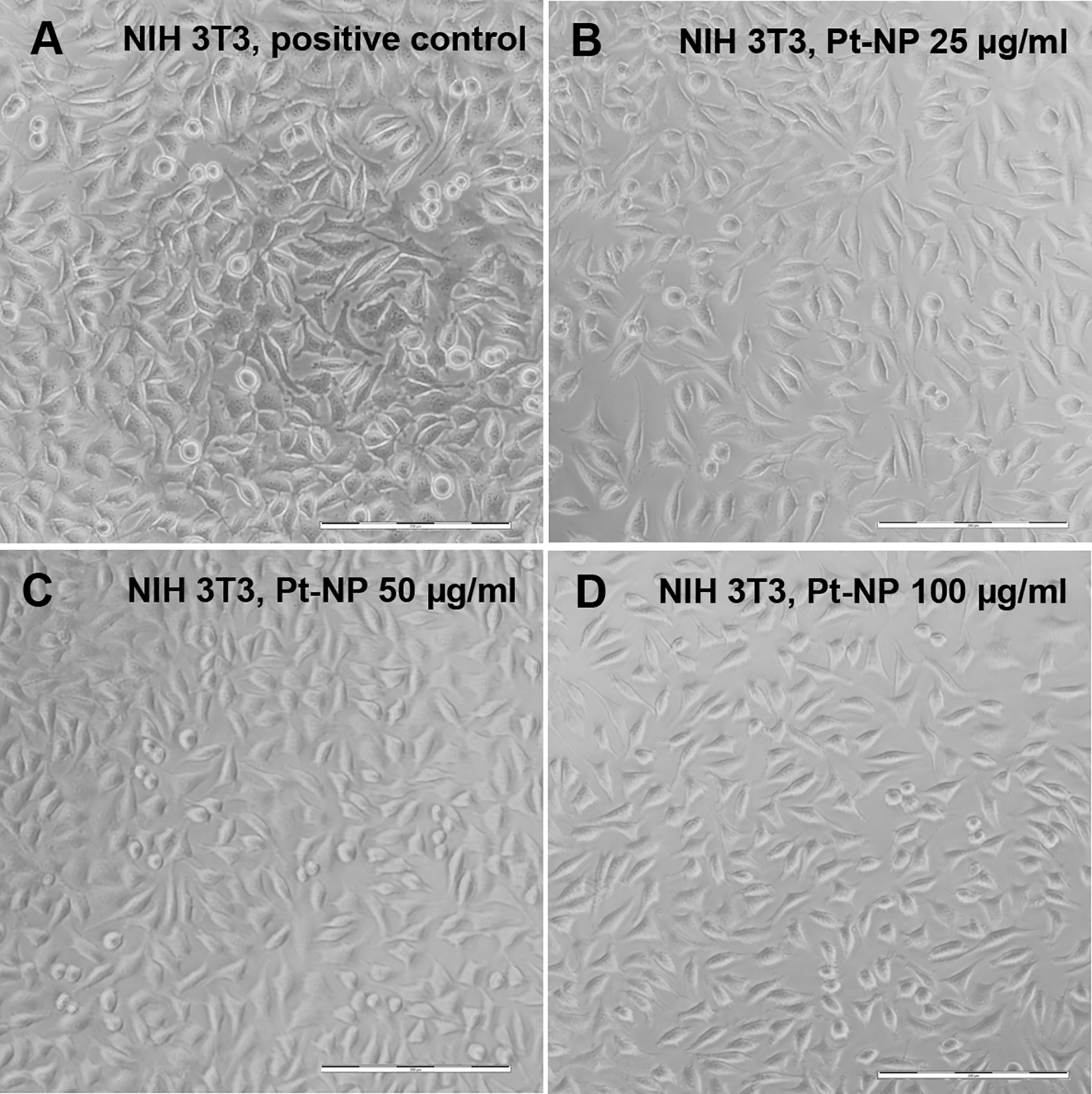
Microscopic characterization of the morphology of NIH 3T3 cells following exposure to Pt-NP. NIH 3T3 cells were cultivated either without any additional Pt particles as reference (**A**) or in culture medium containing 25 μg/ml (**B**), 50 μg/ml (**C**) and 100 μg/ml (**D**) of the Pt-NP. The images demonstrated highly uniform cell adhesion without any morphological impairment throughout the cell cultures assays with varying Pt-NP concentrations. Size of bars: 200 μm.

Since it was assumed that the electrolyte may contain not only ionic Pt, but also Pt particles following electrical stimulation, Pt-NP in concentrations from 5 μg/ml up to 100 μg/ml were added to the cell culture assays 24 h following cell seed. Fig 9 shows a representative microscopic view of the cell morphology of NIH 3T3 cells following Pt-NP exposure. Normal cell adhesion and growth could be observed up to 50 μg/ml Pt-NP. At 100 μg/ml Pt-NP the cell number appeared slightly reduced.

**Fig 10:**
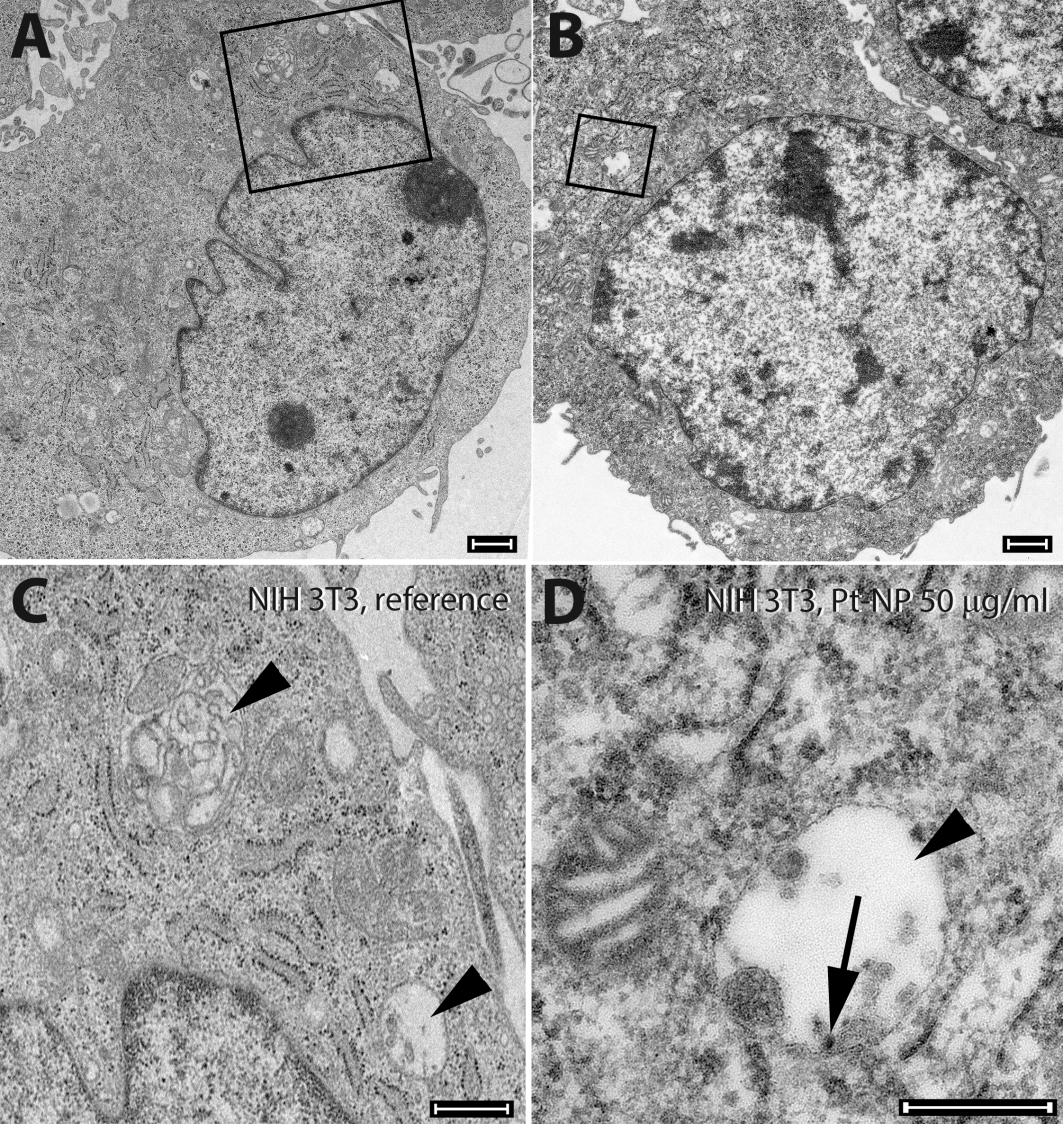
Ultrastructure of NIH 3T3 cells following exposure to Pt-NP. NIH 3T3 cells were cultivated either without any additional Pt particles as reference (**A,** insert in **C**) or in culture medium containing 50 μg/ml (**B,** insert in **D**) of the Pt-NP. No cytotoxic effect of Pt-NP could be found. Fibroblasts were highly active in endocytosis (rectangles in **A** and **B**). In the multi-vesicular bodies (arrowheads in **C** and **D**) accumulation of adsorbed material such as Pt-NP (arrow in **D**) was detected. Size of bars: 1 μm.

After incubation with 5–50 μg/ml Pt-NP NIH 3T3 cells revealed no cytotoxic effect (Fig 10). However, these cells endocytozed the electron-dense nanoparticles in a concentration-dependent manner (Fig 10B and 10D). In particular, the Pt-NP accumulated in the multi-vesicular bodies (Fig 10D). At 100 μg/ml Pt-NP early signs of necrosis, i.e. mitochondrial swelling, were recognized in the cells.

**Fig 11:**
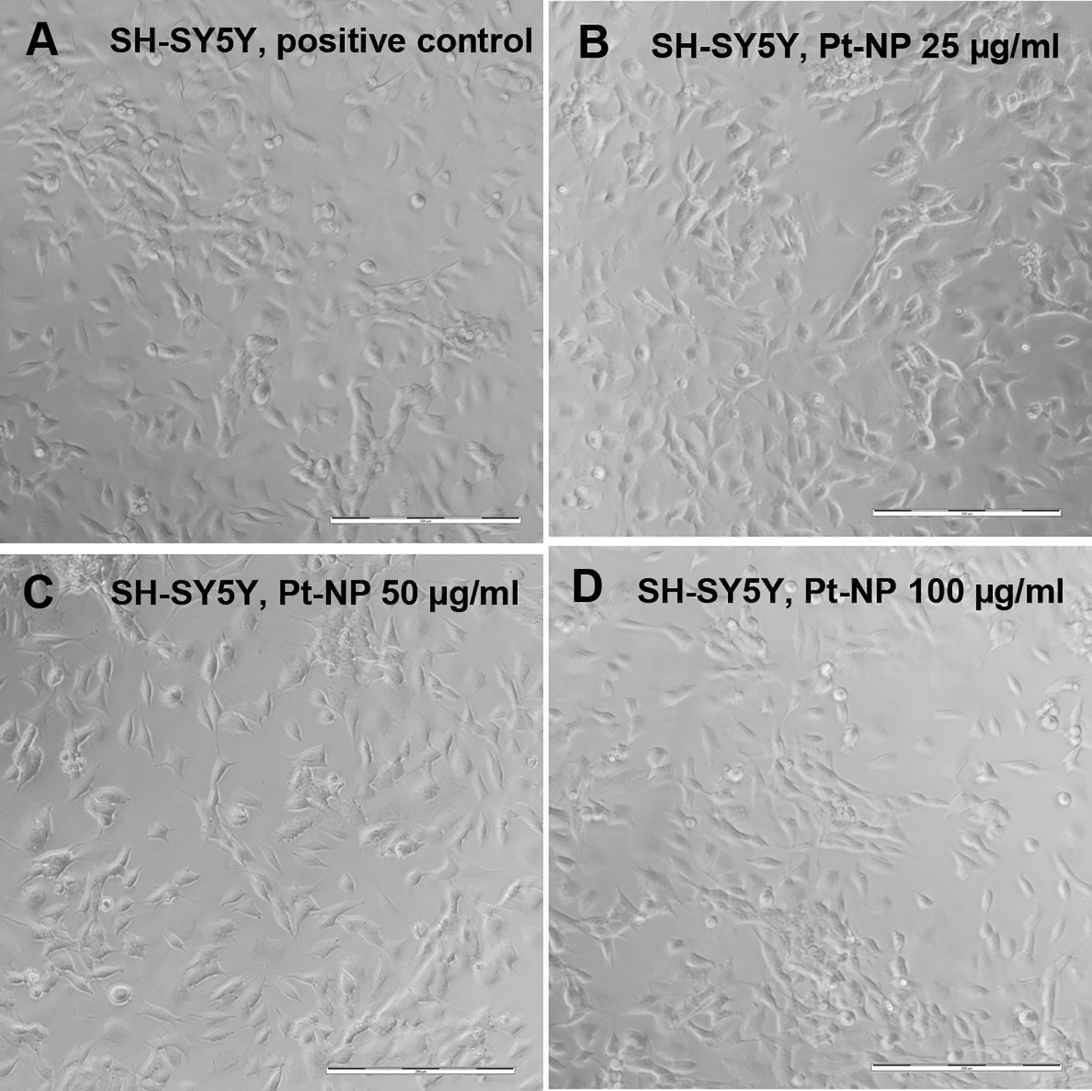
Microscopic characterization of the morphology of SH-SY5Y cells following exposure to Pt-NP. SH-SY5Y cells were cultivated either without any additional Pt particles as reference (**A**) or in culture medium containing 25 μg/ml (**B**), 50 μg/ml (**C**) and 100 μg/ml (**D**) of the Pt-NP. Morphology and adhesion behavior of the SH-SY5Y cells did not change throughout the cultivation assays at varying Pt-NP concentrations. Size of bars: 200 μm.

**Fig 12:**
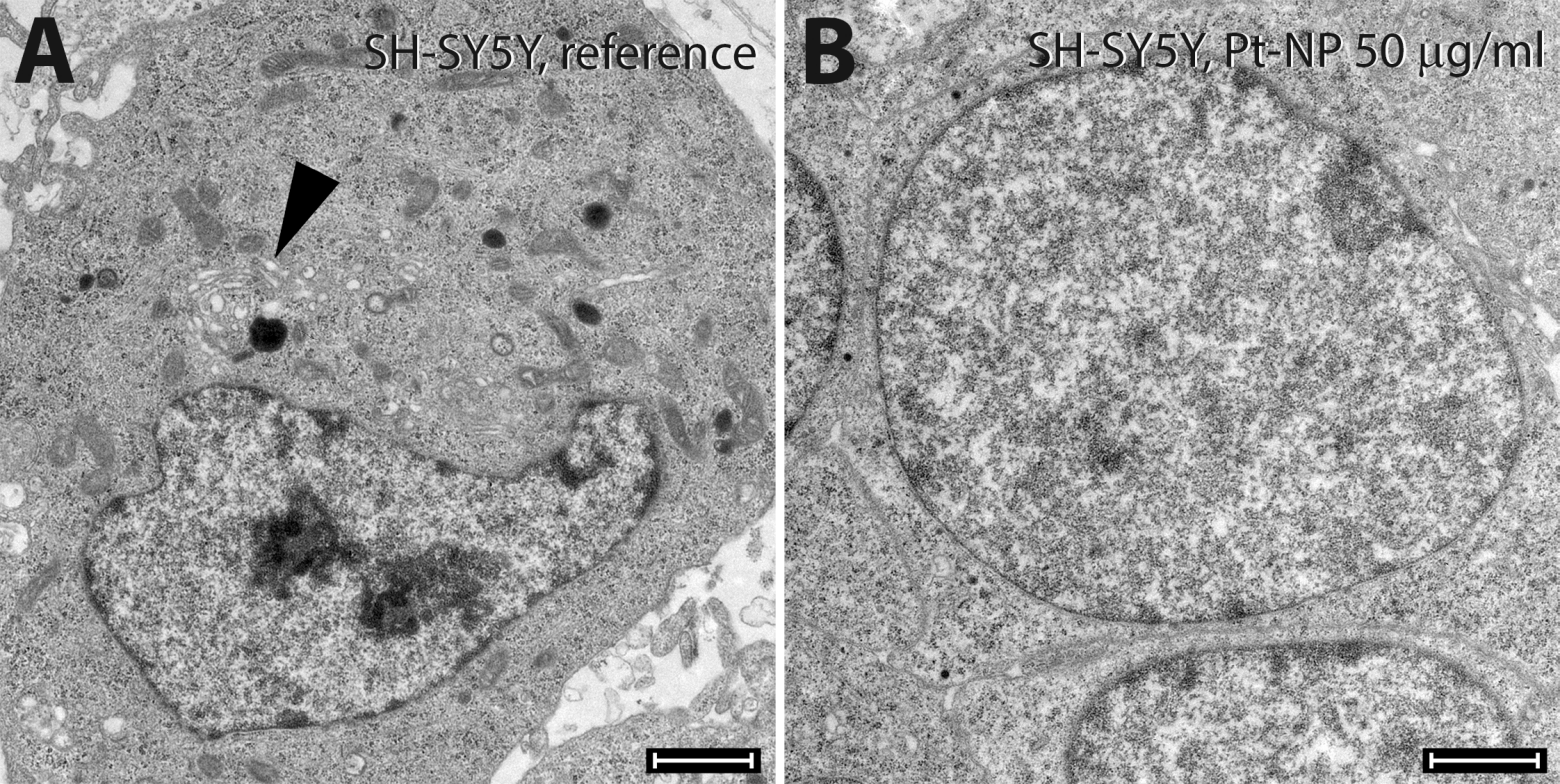
Ultrastructural morphology of SH-SY5Y cells following exposure to Pt-NP. SH-SY5Y cells were cultivated either without any additional Pt particles as reference (**A**) or in culture medium containing 50 μg/ml (**B**) of the Pt-NP. The biosynthesis of synaptic granules as seen in the control cells around the Golgi apparatus (arrowhead in **A**) is typical for those cells; no multi-vesicular bodies of the endocytosis were detected. Neuroblastoma cells in contact with Pt-NP were active only in the secretory pathway. No Pt-NP were evident inside the cell. Size of bars: 200 μm.

The viability of the SH-SY5Y cells was not adversely affected following exposure to Pt-NP at concentrations up to 100 μg/ml (Fig 11). As observed by TEM the neuroblastoma cells showed normal cellular ultrastructure up to 50 μg/ml Pt-NP in the culture medium (Fig 12B). Interestingly, the SH-SY5Y cells exhibited virtually no endocytosis (Fig 12A and 12B). Therefore, no multi-vesicular bodies could be seen in the control cells (Fig 12A) or in cells exposed to Pt-NP at all concentrations used in this study. As shown in Fig 12B, the biosynthesis of synaptic granules remained unchanged in cell cultivation assays supplemented with 50 μg/ml Pt-NP (Fig 12B). However, Pt-NP at a concentration of 100 μg/ml induced mitochondrial disintegration as well as swelling of the endoplasmic reticulum indicating cytotoxic effects of Pt.

**Fig 13:**
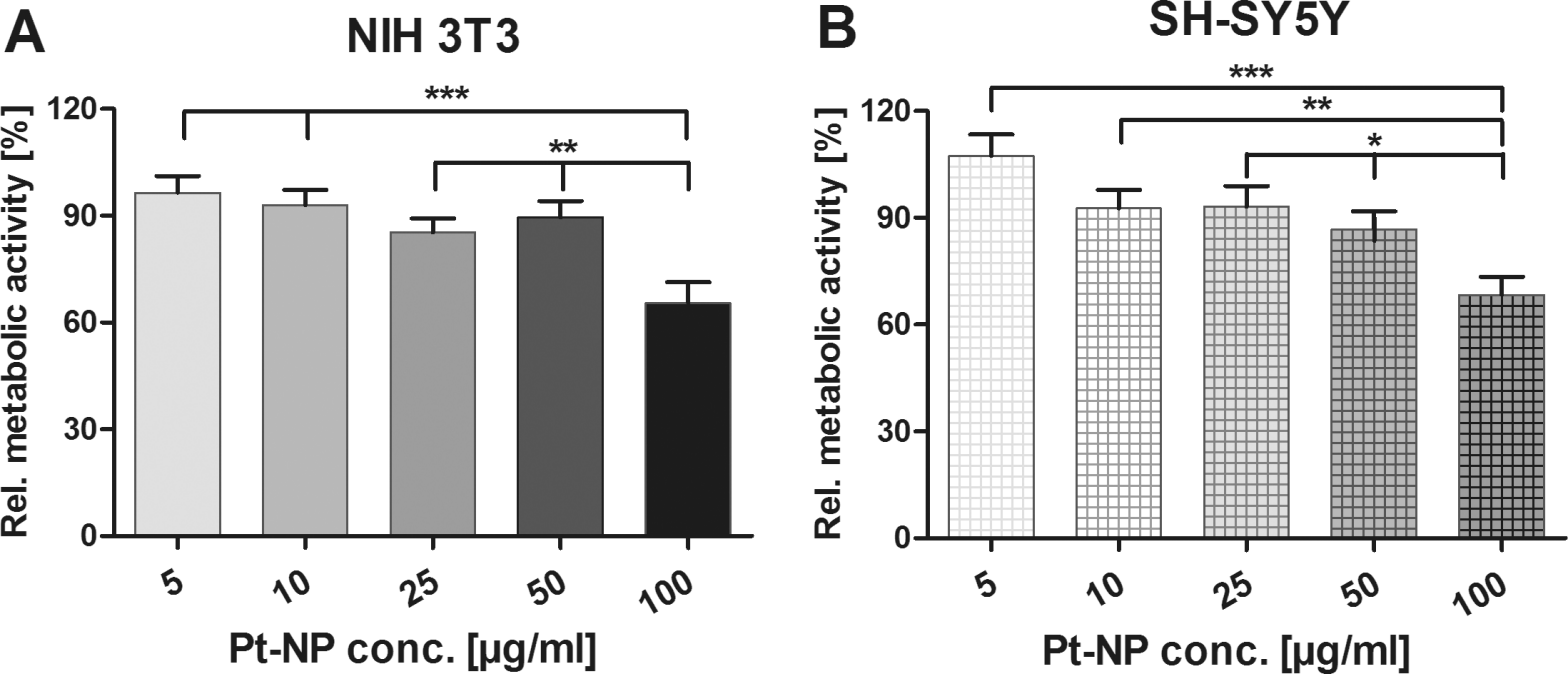
Determination of the effects of Pt-NP with varying concentrations in cell cultivation. Metabolic activity of both NIH 3T3 (**A**) and SH-SY5Y cells (**B**) grown in culture medium supplied with 5 μg/ml– 100 μg/ml Pt-NP was determined by indirect reduction of WST-1 by mitochondrial dehydrogenases to a formazan dye. Optical densities (OD) were measured in 48 h and 6 d cultivation assays (NIH 3T3, n = 12–14; SH-SY5Y, n = 11–15). The resulting formazan dye intensities were related to those obtained from the reference and calculated as a percentage [%]. Each data point is presented as mean and SE_M_. ANOVA with Newman-Keuls multiple comparison test was performed for statistical assessment (***p ≤ 0.001, **p ≤ 0.01, *p ≤ 0.05).

As shown in Fig 13, the metabolic assay with WST-1 revealed no significant influence of the Pt-NP concentrations 5 μg/ml, 10 μg/ml, 25 μg/ml and 50 μg/ml in NIH 3T3 fibroblasts (96.44% ± 4.69, 92.94% ± 4.26, 85.40% ± 3.89 and 89.54% ± 4.66) as well as in neuroblastoma cells (107.3% ± 6.12, 92.61% ± 5.19, 93.16% ± 5.80 and 86.77% ± 5.07), even though a slight downward trend in metabolic activity was observed. By contrast, the administration of 100 μg/ml Pt-NP to the cultivation assays resulted in a significant decrease in cellular activity of both NIH 3T3 (65.42% ± 5.91) and SH-SY5Y cells (68.35% ± 5.07). In accordance with ISO 10993–5 [48], this Pt-NP concentration was deemed cytotoxic (Fig 13).

## Discussion

For the first time, this study presents data on potential neuronal cell damage by Pt derived from electrical stimulation of a human CI electrode in a concentration-dependent manner.

At first, to determine whether the lower NaCl content had any adverse influence of on NIH 3T3 and SH-SY5Y cell metabolism, both cell lines were exposed to 5.93–6.26 mg/ml NaCl. WST assay data and microscopical control of the NIH 3T3 cells allowed to rule out negative biological effects due to the reduced NaCl concentration and medium osmolarity. Despite a greater sensitivity of the SH-SY5Y cells to the lower NaCl content in the cell culture medium, cytotoxic effects could be also excluded as determined by the WST-1 assays.

The Pt-Diss solution used in this study may contain a mixture of both Pt particles of various sizes and shapes, and Pt ions or complexes as also reported in numerous studies [8, 15, 32–34]. Since ICP-MS only enables analysis of the Pt-mass content in total, it is not possible to estimate the contribution of individual components to the cytotoxicity. Nevertheless, our findings demonstrated strong reduction of mitochondrial activity and, thus, induction of oxidative stress following supplementation of the culture medium with Pt-Diss in a concentration-dependent manner. The small contribution of the lower NaCl content in the SH-SY5Y cell culture has to be considered as described above. Stress induction resulting in cell death by incubation of Pt(I-IV) compounds has been described in numerous studies. Only the lethal dosage may differ depending on tissue type, its resistance in cellular uptake of the Pt ions and effective nuclear repair mechanisms of the cells [25–29]. Exposure of 0.5 mM PtCl_4_ to A549 and HaCaT cells and 10 μM cisplatin to HT29 cells induced severe DNA damage and subsequent cell death as the consequence of drastic increase of intracellular reactive oxygen species and lipid peroxidation levels [36, 44]. As previously reviewed, Pt complexes form stable covalent bonds with nuclear DNA., Guanine, in particular, is a susceptible target at the 7-nitrogen atom to be able to form inter- and intrastrand crosslinks [30–31].

The present study demonstrated mitochondrial swelling in both NIH 3T3 and SH-SY5Y cells, which corresponds to the reduction in metabolic activity in the presence of ≥1.64 μg/ml Pt-Diss. Indeed, previous studies found that Pt^2+^ ions accumulate in mitochondria. It follows that cytotoxicity and genotoxicity may be mediated by Pt^2+^ ions [37, 49]. This assumption was supported by Pelka et al. [40] who observed DNA strand breaks in human colon carcinoma (HT29) cells. Although no significant reactive oxygen formation occurred, the authors concluded that Pt ions could be the cause.

To consider the potential biological effects of solid Pt particles contained in the electrolyte, the biocompatibility of Pt-NP 3 nm in size on both cell lines was determined. Additionally, the cellular uptake of Pt-NP and their effects on the ultrastructure were investigated by means of TEM. Furthermore, the particle size chosen allowed their localization inside the cells to be detected by TEM. We found no significant influence of increasing Pt-NP concentrations between 5 μg/ml and 50 μg/ml on either ultrastructure or the metabolism of NIH 3T3 and SH-SY5Y cells. Only at 100 μg/ml Pt-NP concentration cell metabolism and mitochondrial activity were reduced. Our results were in line with previous *in vitro* studies exposing murine L929 fibroblasts, several human cell lines such as RAW264, A549, HaCaT, IMR90 and U251 to Pt-NP with a primary size less than 10 nm: no or only moderate cell death induction were observed [35–38]. Significant toxicity was only found in IMR90, U251 L929 and RAW264 cells following exposure to 80–100 μg/ml Pt-NP [35, 38–39]. *In vivo* administration of 3–10 nm Pt-NP to zebrafish embryos showed dosage dependent mortality rate, hatching delay, phenotypic defects and metal accumulation at concentrations above 50 μg/ml [50]. However, another *in vivo* study reported inhibitory effects on cardiac rate of zebrafish embryos induced by Pt-NP at lower concentrations around 25 μg/ml [42].

Even though particles below 10 nm in size may enable nuclear penetration [36–37, 44], TEM imaging revealed cellular uptake of most of the Pt-NP into the lysosomes, but not into the nuclei and mitochondria of the examined cell lines. It is suggested that either endocytosis or phagocytosis play key roles in cellular uptake of Pt-NP into mammalian cells [36–38, 51–52]. Another study demonstrated both diffusion and endocytosis of Pt-NP into the cytosol of IMR90 and U251 cells depending on the size of the nanoparticles. Electron micrographs revealed agglomerates of Pt-NP in the endosomal-lysosomal compartment, whereas single nanoparticles with sizes limited to less than 10 nm were able to diffuse into the cytosol [35]. Our TEM imaging also demonstrated dose-dependent internalisation of Pt-NP into the multi-vesicular bodies of the NIH 3T3 cells, but no diffusion into the cytosol or nucleus. Interestingly, no Pt-NP were internalized into the SH-SY5Y cells. The Pt-NP used in this study were administered to the cell culture assays as colloidal solution stabilized with polyvinylpyrrolidone (PVP) for effective dispersion. This is well known not only as a nontoxic and bioinert compound [53], but also as a means of protecting metallic nanoparticles from growing and agglomerating [54–55]. Therefore, as expected, Pt-NP dispersion did not form large aggregates accumulating on the surface of both NIH 3T3 and SH-SY5Y cells, as revealed by TEM analysis. However, protein adsorption, especially of medium components such as serum proteins and salts, may significantly enlarge the size of the Pt-NP forming ‘secondary’ nanoparticles, and influence cellular uptake [36]. Admittedly, it has been suggested that those adsorbed proteins are digested in phagolysosomes leaving the nanoparticles ‘naked’ [51]. Nevertheless, the size of the secondary Pt-NP following dispersion in FBS-medium affected neither endocytosis-mediated uptake nor biological activities as compared with the tenfold smaller primary particles [36]. Hence, we assume that dispersion of the commercial Pt-NP colloids in supplemented cell culture medium did not falsify our findings.

Cytotoxicity of Pt was not only found after incubation with ≥1.64 μg/ml Pt-Diss, but also at 100 μg/ml Pt-NP. However, no internalization of the Pt-NP was found in the SH-SY5Y cells prompting the question of whether Pt^2+^ ions could trigger the decrease in mitochondrial activity in SH-SY5Y cells as well. The surface of not only Pt electrodes but also of Pt-NP could corrode, releasing the cytotoxic Pt^2+^ ions into the medium [37, 40, 49].

Based on our results, we assumed that the contribution of the metallic Pt particles in the electrolyte to cell damage may be negligible. Instead, the content of Pt ions and complexes seems to be the main factor inducing oxidative stress and subsequent cell death. By our data disturbations of the cell metabolism in both NIH 3T3 and SH-SY5Y cells appeared *in vitro* at Pt-Diss concentrations between 1.64 μg/ml and 4.1 μg/ml. Previous *in vivo* studies demonstrated low levels of dissolved Pt ranging from 5 ng/ml to 1 μg/ml depending on the electrical-stimulation parameters [17, 24]. In comparison with our data, those findings indicate that corrosion of the electrode contacts may not result in tissue damage. Additionally, diffusion of the corrosion products within the inner ear via the lymphatic fluids may dilute the concentration of toxic Pt compounds, so that the potential of Pt ions or particles to harm living cells would be negligible. Furthermore, new formation of metallic Pt particles may hypothetically function as scavenger of reactive oxygen species to a certain extent [56–59], protecting the tissue from toxic effects of ionic Pt. However–as shown in the present study and reported by several authors, and depending on the cell type and organism–Pt-NP induced oxidative stress, inflammation or other negative biological effects in a concentration-depending manner [39–43]. It is assumed that endocytosis-mediated internalization of the Pt particles results in etching of the surface of the metal particles and their oxidation to ionic Pt due to exposure to low pH and hydrolytic enzymes in the endosomes. Subsequent reduction of the Pt^2+^ ions by H_2_O_2_ generated in the mitochondria may allow the new formed Pt particles to start a new cycle [40, 50]. Consequently, Pt particles may indirectly contribute to cytotoxicity and genotoxicity [37, 49]. In particular, long-term electrical stimulation over several years may cause Pt particles to accumulate in the cell, thus enhancing their cytotoxicity. Furthermore, the complex interactions between the corrosion products and the tissues of the auditory nerve at the Pt/lymphatic fluid interface require more attention. It also has to be considered that, in particular, children need to benefit from the CI throughout their lifetime, so that limitation of Pt corrosion should be of great clinical interest.

Recently, Piret et al., 2015 [60] reported that 3D-nanostructured boron doped diamond (BDD) may be the electrode material of choice for long-term neural implants. The electrode contacts consisted of vertically aligned carbon nanotubes inter-layer template encapsulated within two BDD nanolayers [60, 61]. It was shown that this novel electrode material not only enabled neural cell attachment, survival and neurite outgrowth, but also demonstrated low impedances and high safe charge injection capacity [60].

## Conclusion

This study is an *in vitro* evaluation of the murine fibroblast NIH 3T3 and human neuroblastoma SH-SY5Y cell culture model exposed to commercially manufactured Pt-NP and corrosion products following electrical stimulation of a human CI electrode (Pt-Diss). We found negligible oxidative stress in both NIH 3T3 and SH-SY5Y cells up to 50 μg/ml Pt-NP, which was in line with several other studies. Interestingly, no diffusion of the very small Pt-NP into the cells was found. In contrast to Pt-NP, corrosion products (Pt-Diss) with platinum mass fractions between 1.64 μg/ml and 8.2 μg/ml induced cell death in both murine and human cell lines. Taken together, our data indicate that corrosion of Pt electrode contacts may not lead to toxic concentrations in the surrounding inner ear tissues, since low levels of dissolved Pt ranging from 5 ng/ml to 1 μg/ml have been previously demonstrated. However, long-term cytotoxicity over a period of several years during Pt electrode corrosion requires investigation, as do the interactions of the corrosion products with the tissues of the auditory nerve at the Pt/lymphatic fluid interface.

## Abbreviations

BSE: Backscattered electrons
CI: cochlear implant
FBS: fetal bovine serum
DIN: German Institute for Standardisation
ICP-MS: inductive coupled plasma-mass spectrometry
OD: optical density
Pt-NP: Pt nanoparticles
Pt-Diss: Pt dissolute
SE_M_: Standard error of mean
SEM: scanning electron microscopy
TEM: transmission electron microscopy
